# Dynamic examination of closed cylindrical shells utilizing the differential transform method

**DOI:** 10.1038/s41598-024-66095-w

**Published:** 2024-07-03

**Authors:** Amir Esmaeel Khosravi, Farzad Shahabian, Ahmad Aftabi Sani

**Affiliations:** Department of Civil Engineering, Faculty of Engineering, Ferdowsi University of Mashhad, Mashhad, Iran

**Keywords:** Differential transform method, Cylindrical shells, Natural frequency, Dimensionless parametric analysis, Civil engineering, Aerospace engineering

## Abstract

This article presents an innovative approach using the Differential Transform Method (DTM) to analyze the vibration characteristics of cylindrical shells, integrating Taylor's series with Sander's classical theory. It demonstrates DTM's efficiency, accuracy, and potential as an alternative method. The study introduces a novel application of the DTM in exploring the free vibration of cylindrical shells, detailing a technique to address challenges such as normalization, linear solution methodologies, and parameter derivative modifications. A dimensionless parameter analysis evaluates the impact of length, radius, thickness, and modulus of elasticity. Comparative analysis with Hybrid Finite Element Method (FEM) data and validation against existing literature highlights DTM's precision and reliability. In conclusion, DTM offers a robust solution for the eigenvalue problem in coupled differential equations, providing accurate vibration parameters. Additionally, an important relationship between the modulus of elasticity and frequency in the dimensionless state was obtained.

## Introduction

Cylindrical shells have been the subject of much research due to their wide range of use in industrial engineering, considering the utilization of such geometric shapes in industry and sensitive components. This type of structure requires a much more detailed and comprehensive investigation. The studies conducted in this field can be categorized into different applications such as pressure vessels, roof structures, open structures, and marine structures. Hence, various theories and solutions have been proposed for the vibration analysis of such structures ^1–4^.

In one of the earliest related studies, Amabili investigated the vibration characteristics of cylindrical shells filled with liquid ^5^. The study examined the impact of water content on frequency data using exact solution, fluid finite elements, and the boundary element method. With the water surface parallel to the shell axis, natural frequencies and mode shapes were determined via the Galerkin equation. The study concluded by comparing modal analysis results for stainless steel with experimental data from various sources.

Zhang et al. studied the vibration of cylindrical shells using the wave propagation method, which works well across different boundary conditions and shell structures, showing good agreement with existing data ^6^. In 2006, Kandasamy et al. introduced a relationship for the free vibration of open circular cylindrical deep shells in flat and inclined configurations ^7^. Using the first-order shear deformation theory, this study accounted for rotational inertia and shear deformation, analyzing shells from thin to thick. The Rayleigh–Ritz method computed frequencies and mode shapes, and a parametric investigation focused on inclined shells for a comprehensive analysis.

Zhang et al. utilized the local adaptive differential quadrature method (LaDQM) to derive a relationship for the frequency of circular cylindrical shells under diverse boundary conditions ^8^. Employing the Goldenweizer-Novozhilov theory of thin shells, this research focused on analyzing and formulating these shells.

Pellicano introduced an analytical approach encompassing both linear and non-linear analyses for circular cylindrical shells under diverse boundary conditions, drawing from the Sander-Koiter theory ^9^. This study focused on parameterizing simple and clamped boundary conditions. The method demonstrated its accuracy and efficacy by spanning a broad frequency range and precisely aligning with data available in related literature. Noteworthy is the method's capability to address linear and non-linear vibrations concurrently. Xing et al. devised an exact solution grounded in the Donnell-Mushtari shell theory to assess the vibration characteristics of circular cylindrical shells under classical boundary conditions ^10^. Employing an exact solution method, this study reevaluated vibration parameters. A departure from previous approaches that included the consideration of shear diaphragms at the edges, a factor eliminated in this investigation.

In a separate study, Duan et al. employed the discrete singular convolution (DSC) method to analyze the vibration characteristics of circular thin plates, exploring both uniform and stepped thickness variations. Notably, this research introduced innovative usage of circular components ^11^. Tornabene et al. conduct a comparative study on vibration parameters using various methods, including finite element methods and generalized differential quadrature (GDQ) ^12^. The study covers single-layer, multi-layer, isotropic, composite, and sandwich shells in cylindrical and spherical geometries. In a related investigation, Qin et al. explore free vibration characteristics in cylindrical shells with varied boundary conditions using analytical methods like Sander’s theory and modified Fourier series ^13^. Artificial springs are introduced to simulate different boundary conditions.

Li et al. investigated the free vibration characteristics of stepped and uniform circular cylindrical shells under arbitrary boundary conditions using a semi-analytical approach ^14^. Their study integrated Flügge theory, employing Fourier series and Jacobi polynomials to represent displacement functions. Additionally, Kashani and Aftabi Sani utilized polar finite elements to explore the dynamic characteristics of horizontal cylindrical shells, accounting for the influence of fluid surface waves ^15^. Their proposed methodology integrated structural shape functions derived from the exact solution, polar finite elements, and Sander’s theory. This investigation encompassed an analysis of structural and fluid interaction effects.

Wang et al. explored vibration analysis employing a three-dimensional exact method for a thick shell situated on a Pasternak foundation ^16^. This methodology described the functions of thick shells as a combination of three-dimensional Fourier cosine series and auxiliary functions. Notably, the improved Fourier series eliminated all discontinuities associated with displacement and their derivatives at the edges, regardless of the imposed boundary conditions. Comparative analysis was performed against ABAQUS data sources, presenting geometrical parametric studies of elastic foundation coefficients and elastic restraint parameters.

Dongxu Du et al. examine free vibration analyses of rotating hard-coating cylindrical shells under varied conditions ^17^. Employing characteristic orthogonal polynomials and considering forces like Coriolis, they establish equations of motion using the Rayleigh–Ritz method. Results, validated against finite element analysis, assess the effects of boundary conditions, hard coating parameters, and rotating speed on vibration behaviors. Talezadehlari uses the Multi-domain Generalized Differential Quadrature (GDQ) method to analyze the free vibration behavior of composite shell/panels with and without central square cutouts ^18^. Results, validated against ABAQUS and literature, assess the influence of cutout presence and size on vibrational behavior under various conditions.

In a seminal work dating back to 1980, Zhou introduced the differential transform method (DTM) as an effective tool for addressing linear and non-linear problems encompassing boundary conditions, initial conditions, and eigenvalues ^19^. This semi-analytical, semi-numerical method relies on Taylor series expansion. Subsequently, numerous researchers have adapted and expanded this method, applying it extensively across various domains, including beams, columns, plates, and shells ^20–23^. This review summarizes the breadth of studies conducted within these domains.

In a related investigation, Yesilce explored the free vibration characteristics of semi-rigid Reddy-Bickford beams positioned on elastic soil in their research ^24^. The beam was situated in two soil regions, and the problem was solved utilizing Hamilton's equations. Additionally, Wattanasakulpong and Ungbhakorn examined the free vibration behavior of Functionally Graded (FG) beams, assessing various boundary conditions and adhering to the energy distribution principle ^25^. Employing the DTM, a method renowned for its reported accuracy and low computational complexity, the study discussed outputs such as mode shapes and frequencies concerning diverse parameters and boundary conditions. Additionally, Rezaiee-Pajand et al. utilized the Euler–Bernoulli theory for their beam analysis ^26^.

Kumar Jena and Chakraverty employed the DTM to explore the free vibration behavior of Nano beams, leveraging the non-local Euler–Bernoulli theory ^27^. Their study delved into the free vibration analysis of Nano beams, encompassing various boundary conditions, utilizing MATLAB software as a computational tool. Rezaiee Pajand et al. investigate the free vibration of a gabled frame with rotational springs ^28^. They use the DTM to derive equations, presenting mode shapes, and validate solutions using finite element analysis. Hang Xu et al. investigate the free vibration of a spinning functionally graded grapheme platelet-reinforced metal foam (FG-GPLRMF) beam ^29^. They extend the DTM to analyze flap-wise and chord wise bending vibrations, revealing influences of GPL characteristics. Rezaee-Pajand et al. analyze the free vibrations of Euler–Bernoulli beams near in viscid, irrotational fluids and Winkler soil using the DTM ^30^. The study assesses various soil-beam-fluid systems, emphasizing differences in supports and soil characteristics. Results are compared with finite element analysis, confirming the method's precision and reliability.

To the best of the author's knowledge, the utilization of the DTM in analyzing vibration parameters of closed cylindrical shells with Sander’s theory remains unexplored. Consequently, the problem was solved via DTM. Additionally, an assessment was conducted on the impact of computational parameters -shell length, thickness, radius, and elasticity modulus- on frequency in a dimensionless manner. This evaluation, alongside the DTM, involved the use of the hybrid finite element method to ascertain the accuracy and efficiency of the proposed method.

## Structural governing differential equation

**Figure 1:**
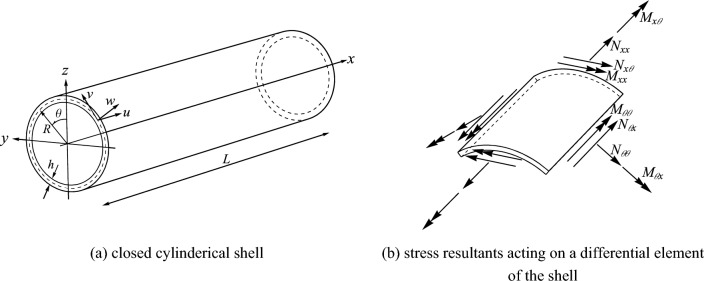
The geometry of closed circular cylindrical shells ^32^. (**a**) Closed cylinderical shell. (**b**) Stress resultants acting on a differential element of the shell.

Fundamental equations for a general closed cylindrical thin shell, as illustrated in Fig. 1, which depicts the geometric configuration of the closed cylindrical, can be established by utilizing the Sander’s thin shell theory. It is notable that the Sander’s shell theory is based on Love’s first approximation in which all the strains for small rigid-body motions vanished ^31^. In addition, the out-of-plane shear forces are eliminated. Thus, the five equilibrium equations in the cylindrical coordinates reduce to three equations. The details of shell are shown in Fig. 1a,b. Finally, by adding the translatory inertia parameters to the right-hand side of the motion equations one can obtain ^32^:

1-1 $$ \frac{{\partial N_{xx} }}{\partial x} + \frac{1}{R}\frac{{\partial N_{x\theta } }}{\partial \theta } - \frac{1}{{2R^{2} }}\frac{{\partial M_{x\theta } }}{\partial \theta } = \rho h\frac{{\partial^{2} u}}{{\partial t^{2} }}, $$

1-2 $$ \frac{1}{R}\frac{{\partial N_{\theta \theta } }}{\partial \theta } + \frac{{\partial N_{x\theta } }}{\partial x} + \frac{3}{2R}\frac{{\partial M_{x\theta } }}{\partial x} + \frac{1}{{R^{2} }}\frac{{\partial M_{\theta \theta } }}{\partial \theta } = \rho h\frac{{\partial^{2} v}}{{\partial t^{2} }}, $$

1-3 $$ \frac{{\partial^{2} M_{xx} }}{{\partial x^{2} }} + \frac{2}{R}\frac{{\partial^{2} M_{x\theta } }}{\partial x\partial \theta } + \frac{1}{{R^{2} }}\frac{{\partial^{2} M_{\theta \theta } }}{{\partial \theta^{2} }} - \frac{{N_{\theta \theta } }}{R} = \rho h\frac{{\partial^{2} w}}{{\partial t^{2} }}, $$

where, $N_{xx}$, $N_{\theta \theta }$ and $N_{x\theta }$ represent the stress resultants per unit length of the shell, and $M_{xx}$, $M_{\theta \theta }$ and $M_{x\theta }$ denote the stress couples per unit length, correspondingly. In addition, the coordinates of the shell are expressed by $x$ and $\theta$, and $u$, $v$ and $w$ stand for the axial, circumferential and radial displacements of the mid-plane of the shell. Besides, $R$ is the shell mean radius, $h$ is its thickness, $\rho$ stands for its material density and $t$ denotes the time.

On the other hand, the strain vector of the mid-plane of the shell can be written as:

2 $$ {{\varvec{\upvarepsilon}}} = \left\{ {\begin{array}{*{20}c} {\begin{array}{*{20}c} {\begin{array}{*{20}c} {\varepsilon_{xx} } & {\varepsilon_{\theta \theta } } \\ \end{array} } & {2\varepsilon_{x\theta } } & {\kappa_{xx} } \\ \end{array} } & {\kappa_{\theta \theta } } & {2\kappa_{x\theta } } \\ \end{array} } \right\}^{{\text{T}}} , $$

in which:

3 $$ \begin{array}{*{20}l} {\varepsilon_{xx} = \frac{\partial u}{{\partial x}},} \hfill & {\,\varepsilon_{\theta \theta } = \frac{1}{R}\frac{\partial v}{{\partial \theta }} + \frac{w}{R},} \hfill & {2\varepsilon_{x\theta } = \frac{\partial v}{{\partial x}} + \frac{1}{R}\frac{\partial u}{{\partial \theta }},} \hfill \\ {\kappa_{xx} = - \frac{{\partial^{2} w}}{{\partial x^{2} }},} \hfill & {\kappa_{\theta \theta } = - \frac{1}{{R^{2} }}\frac{{\partial^{2} w}}{{\partial \theta^{2} }} + \frac{1}{{R^{2} }}\frac{\partial v}{{\partial \theta }},} \hfill & {2\kappa_{x\theta } = - \frac{2}{R}\frac{{\partial^{2} w}}{\partial x\partial \theta } + \frac{3}{2R}\frac{\partial v}{{\partial x}} - \frac{1}{{2R^{2} }}\frac{\partial u}{{\partial \theta }}.} \hfill \\ \end{array} $$

Besides, the corresponding stress vector can be expressed as:

4 $$ {{\varvec{\upsigma}}} = \left\{ {\begin{array}{*{20}c} {\begin{array}{*{20}c} {\begin{array}{*{20}c} {N_{xx} } & {N_{\theta \theta } } \\ \end{array} } & {N_{x\theta } } & {M_{xx} } \\ \end{array} } & {M_{\theta \theta } } & {M_{x\theta } } \\ \end{array} } \right\}^{{\text{T}}} . $$

Subsequently, the following constitutive equation relates the strain and stress components:

5-1 $$ {{\varvec{\upsigma}}}\,{ = }\,\left\{ {\begin{array}{*{20}c} {N_{xx} } \\ {N_{\theta \theta } } \\ {N_{x\theta } } \\ {M_{xx} } \\ {M_{\theta \theta } } \\ {M_{x\theta } } \\ \end{array} } \right\} = {\mathbf{P\varepsilon }} = \left\{ {\begin{array}{*{20}c} {D(\varepsilon_{xx} + \mu \varepsilon_{\theta \theta } )} \\ {D(\varepsilon_{\theta \theta } + \mu \varepsilon_{xx} )} \\ {D(1 - \mu )\varepsilon_{x\theta } } \\ {\eta (\kappa_{xx} + \mu \kappa_{\theta \theta } )} \\ {\eta (\kappa_{\theta \theta } + \mu \kappa_{xx} )} \\ {\eta (1 - \mu )\kappa_{x\theta } } \\ \end{array} } \right\} $$

5-2 $$ D = \frac{Eh}{{1 - \mu^{2} }} $$

5-3 $$ \eta = \frac{{Eh^{3} }}{{12(1 - \mu^{2} )}} $$

in which, the elements $p_{ij}$ in the elasticity matrix, $P$, contains the mechanical properties of the shell material. In the next step, substituting Eq. (3) into Eq. (2) one can obtain the strain vector of the shell as a function of mid-plane shell displacements. Afterwards, with the aid of Eqs. (4) and (5), the governing differential equations of the cylinder, i.e. Equation (1), takes the following form in terms of $u$, $v$ and $w$:

6-1 $$ \begin{gathered} R^{2} \frac{{\partial^{2} u}}{{\partial x^{2} }} + \frac{(1 + \mu )R}{2}\frac{{\partial^{2} v}}{\partial x\partial \theta } + \mu R\frac{\partial w}{{\partial x}} + \frac{(1 - \mu )}{2}\frac{{\partial^{2} u}}{{\partial \theta^{2} }} \hfill \\ + \beta (1 - \mu )\left( {\frac{R}{2}\frac{{\partial^{3} w}}{{\partial x\partial \theta^{2} }} - \frac{3R}{8}\frac{{\partial^{2} v}}{\partial x\partial \theta } + \frac{1}{8}\frac{{\partial^{2} u}}{{\partial \theta^{2} }}} \right) = \frac{\rho h}{\alpha }\frac{{\partial^{2} u}}{{\partial t^{2} }}, \hfill \\ \end{gathered} $$

6-2 $$ \begin{gathered} \frac{{\partial^{2} v}}{{\partial \theta^{2} }} + \frac{\partial w}{{\partial \theta }} + \frac{(1 + \mu )R}{2}\frac{{\partial^{2} u}}{\partial x\partial \theta } + \frac{{(1 - \mu )R^{2} }}{2}\frac{{\partial^{2} v}}{{\partial x^{2} }} + \beta \,\left( { - \frac{{(3 - \mu )R^{2} }}{2}\frac{{\partial^{3} w}}{{\partial x^{2} \partial \theta }} + \frac{{9(1 - \mu )R^{2} }}{8}\frac{{\partial^{2} v}}{{\partial x^{2} }}} \right. \hfill \\ \left. { - \frac{3R(1 - \mu )}{8}\frac{{\partial^{2} u}}{\partial x\partial \theta } - \frac{{\partial^{3} w}}{{\partial \theta^{3} }} + \frac{{\partial^{2} v}}{{\partial \theta^{2} }}} \right) = \frac{\rho h}{\alpha }\frac{{\partial^{2} v}}{{\partial t^{2} }}, \hfill \\ \end{gathered} $$

6-3 $$ \begin{gathered} \beta \,\left( { - R^{4} \frac{{\partial^{4} w}}{{\partial x^{4} }} - 2R^{2} \frac{{\partial^{4} w}}{{\partial x^{2} \partial \theta^{2} }} + \frac{{(3 - \mu )R^{2} }}{2}\frac{{\partial^{3} v}}{{\partial x^{2} \partial \theta }}} \right.\left. { - \frac{(1 - \mu )R}{2}\frac{{\partial^{3} u}}{{\partial x\partial \theta^{2} }} - \frac{{\partial^{4} w}}{{\partial \theta^{4} }} + \frac{{\partial^{3} v}}{{\partial \theta^{3} }}} \right) \hfill \\ - \frac{\partial v}{{\partial \theta }} - w - \mu R\frac{\partial u}{{\partial x}} = \frac{\rho h}{\alpha }\frac{{\partial^{2} w}}{{\partial t^{2} }}, \hfill \\ \end{gathered} $$

in which the parameter $\alpha$ and the nondimensional parameter $\beta$ are defined as:

7-1 $$ \alpha = \frac{Eh}{{R^{2} (1 - \mu^{2} )}}, $$

7-2 $$ \beta = \frac{{h^{2} }}{{12R^{2} }}, $$

herein, $\mu$ represents the Poisson’s ratio and $E$ stands for Young’s modulus. It is noteworthy that the parameters $u$, $v$ and $w$ are functions of the system coordinate and time. In the next stage, the well-known Fourier transform with the following definition is employed to eliminate the time dependence of governing motion equations of the cylinder.

8 $$ f^{ * } (\omega ) = \int_{ - \infty }^{ + \infty } {f(t) \, } {\text{e}}^{{{\text{ - i}}\omega t}} {\text{d}}t, $$

where $f^{ * } (\omega )$ is the corresponding Fourier transformed of the time-continuous function $f(t)$. In addition, ${\text{i}}$ is the unit imaginary number (${\text{i}}^{2} = - 1$) and $\omega$ denotes the circular frequency. Now, applying the mentioned transformation on both sides of relations of Eq. (6) gives:

9-1 $$ \begin{gathered} {\text{F}}\left[ {R^{2} \frac{{\partial^{2} u}}{{\partial x^{2} }} + \frac{(1 + \mu )R}{2}\frac{{\partial^{2} v}}{\partial x\partial \theta } + \mu R\frac{\partial w}{{\partial x}} + \frac{(1 - \mu )}{2}\frac{{\partial^{2} u}}{{\partial \theta^{2} }}} \right. \hfill \\ \left. { + \beta (1 - \mu )\left( {\frac{R}{2}\frac{{\partial^{3} w}}{{\partial x\partial \theta^{2} }} - \frac{3R}{8}\frac{{\partial^{2} v}}{\partial x\partial \theta } + \frac{1}{8}\frac{{\partial^{2} u}}{{\partial \theta^{2} }}} \right)} \right] = {\text{F}}\left[ {\frac{\rho h}{\alpha }\frac{{\partial^{2} u}}{{\partial t^{2} }}} \right], \hfill \\ \end{gathered} $$

9-2 $$ \begin{gathered} {\text{F}}\left[ {\frac{{\partial^{2} v}}{{\partial \theta^{2} }} + \frac{\partial w}{{\partial \theta }} + \frac{(1 + \mu )R}{2}\frac{{\partial^{2} u}}{\partial x\partial \theta } + \frac{{(1 - \mu )R^{2} }}{2}\frac{{\partial^{2} v}}{{\partial x^{2} }} + \beta \,\left( { - \frac{{(3 - \mu )R^{2} }}{2}\frac{{\partial^{3} w}}{{\partial x^{2} \partial \theta }} + \frac{{9(1 - \mu )R^{2} }}{8}\frac{{\partial^{2} v}}{{\partial x^{2} }}} \right.} \right. \hfill \\ \left. {\left. { - \frac{3R(1 - \mu )}{8}\frac{{\partial^{2} u}}{\partial x\partial \theta } - \frac{{\partial^{3} w}}{{\partial \theta^{3} }} + \frac{{\partial^{2} v}}{{\partial \theta^{2} }}} \right)} \right] = {\text{F}}\left[ {\frac{\rho h}{\alpha }\frac{{\partial^{2} v}}{{\partial t^{2} }}} \right], \hfill \\ \end{gathered} $$

9-3 $$ \begin{gathered} {\text{F}}\left[ {\beta \,\left( { - R^{4} \frac{{\partial^{4} w}}{{\partial x^{4} }} - 2R^{2} \frac{{\partial^{4} w}}{{\partial x^{2} \partial \theta^{2} }} + \frac{{(3 - \mu )R^{2} }}{2}\frac{{\partial^{3} v}}{{\partial x^{2} \partial \theta }}} \right.\left. { - \frac{(1 - \mu )R}{2}\frac{{\partial^{3} u}}{{\partial x\partial \theta^{2} }} - \frac{{\partial^{4} w}}{{\partial \theta^{4} }} + \frac{{\partial^{3} v}}{{\partial \theta^{3} }}} \right)} \right. \hfill \\ \left. { - \frac{\partial v}{{\partial \theta }} - w - \mu R\frac{\partial u}{{\partial x}}} \right] = {\text{F}}\left[ {\frac{\rho h}{\alpha }\frac{{\partial^{2} w}}{{\partial t^{2} }}} \right], \hfill \\ \end{gathered} $$

which results in:

10-1 $$ \begin{gathered} R^{2} \frac{{\partial^{2} u^{ * } }}{{\partial x^{2} }} + \frac{(1 + \mu )R}{2}\frac{{\partial^{2} v^{*} }}{\partial x\partial \theta } + \mu R\frac{{\partial w^{*} }}{\partial x} + \frac{(1 - \mu )}{2}\frac{{\partial^{2} u^{*} }}{{\partial \theta^{2} }} \hfill \\ + \beta (1 - \mu )\left( {\frac{R}{2}\frac{{\partial^{3} w^{*} }}{{\partial x\partial \theta^{2} }} - \frac{3R}{8}\frac{{\partial^{2} v^{*} }}{\partial x\partial \theta } + \frac{1}{8}\frac{{\partial^{2} u^{*} }}{{\partial \theta^{2} }}} \right) = - \frac{{\rho h\omega^{2} }}{\alpha }u^{*} , \hfill \\ \end{gathered} $$

10-2 $$ \begin{gathered} \frac{{\partial^{2} v^{*} }}{{\partial \theta^{2} }} + \frac{{\partial w^{*} }}{\partial \theta } + \frac{(1 + \mu )R}{2}\frac{{\partial^{2} u^{*} }}{\partial x\partial \theta } + \frac{{(1 - \mu )R^{2} }}{2}\frac{{\partial^{2} v^{*} }}{{\partial x^{2} }} + \beta \,\left( { - \frac{{(3 - \mu )R^{2} }}{2}\frac{{\partial^{3} w^{*} }}{{\partial x^{2} \partial \theta }} + \frac{{9(1 - \mu )R^{2} }}{8}\frac{{\partial^{2} v^{*} }}{{\partial x^{2} }}} \right. \hfill \\ \, \left. { - \frac{3R(1 - \mu )}{8}\frac{{\partial^{2} u^{*} }}{\partial x\partial \theta } - \frac{{\partial^{3} w^{*} }}{{\partial \theta^{3} }} + \frac{{\partial^{2} v^{*} }}{{\partial \theta^{2} }}} \right) = - \frac{{\rho h\omega^{2} }}{\alpha }v^{*} , \hfill \\ \end{gathered} $$

10-3 $$ \begin{gathered} \beta \,\left( { - R^{4} \frac{{\partial^{4} w^{*} }}{{\partial x^{4} }} - 2R^{2} \frac{{\partial^{4} w^{*} }}{{\partial x^{2} \partial \theta^{2} }} + \frac{{(3 - \mu )R^{2} }}{2}\frac{{\partial^{3} v^{*} }}{{\partial x^{2} \partial \theta }}} \right.\left. { - \frac{(1 - \mu )R}{2}\frac{{\partial^{3} u^{*} }}{{\partial x\partial \theta^{2} }} - \frac{{\partial^{4} w^{*} }}{{\partial \theta^{4} }} + \frac{{\partial^{3} v^{*} }}{{\partial \theta^{3} }}} \right) \hfill \\ - \frac{{\partial v^{*} }}{\partial \theta } - w^{*} - \mu R\frac{{\partial u^{*} }}{\partial x} = - \frac{{\rho h\omega^{2} }}{\alpha }w^{*} , \hfill \\ \end{gathered} $$

where $u^{*} (x,\theta )$, $v^{*} (x,\theta )$ and $w^{*} (x,\theta )$ refer to the Fourier transformed of displacement functions $u(x,\theta ,t)$, $v(x,\theta ,t)$ and $w(x,\theta ,t)$, respectively. It is noteworthy to mention that the inverse Fourier transform can be obtained as:

11 $$ f(t) = \frac{1}{{2{\uppi }}}\int_{ - \infty }^{ + \infty } {f^{ * } (\omega ) \, } {\text{e}}^{{{\text{i}}\omega t}} {\text{d}}\omega . $$

It should be mentioned that the cylinder in the present study is assumed to be freely simply supported at both ends which means they are constrained by a very thin diaphragm. Thereupon, the following conditions should be satisfied at both curved edges:

12 $$ N_{xx} = M_{xx} = v = w = 0\,\,\, {\text{at}}\,\,\,\,x = 0,L. $$

Consequently, the following displacement functions that meet the aforementioned boundary conditions are assumed:

13 $$ \left\{ {\begin{array}{*{20}l} {u^{*} (x,\theta ) = \hat{U}(\theta ) \times \cos \frac{{m{\uppi }x}}{L},} \hfill \\ {v^{*} (x,\theta ) = \hat{V}(\theta ) \times \sin \frac{{m{\uppi }x}}{L},} \hfill \\ {w^{*} (x,\theta ) = \hat{W}(\theta ) \times \sin \frac{{m{\uppi }x}}{L},} \hfill \\ \end{array} } \right. $$

where $m$ is the axial wave number. Finally, substituting Eq. (13) into the relations of Eq. (10) yield:

14-1 $$ \begin{gathered} - \lambda_{m}^{2} \hat{U}(\theta ) + \frac{1}{2}\lambda_{m} (1 + \mu )\frac{{{\text{d}}\hat{V}(\theta )}}{{{\text{d}}\theta }} + \lambda_{m} \mu \hat{W}(\theta ) + \frac{(1 - \mu )}{2}\frac{{{\text{d}}^{2} \hat{U}(\theta )}}{{{\text{d}}\theta^{2} }} \hfill \\ \, + \beta (1 - \mu )\left( {\frac{1}{2}\lambda_{m} \frac{{{\text{d}}^{2} \hat{W}(\theta )}}{{{\text{d}}\theta^{2} }} - \frac{3}{8}\lambda_{m} \frac{{{\text{d}}\hat{V}(\theta )}}{{{\text{d}}\theta }} + \frac{1}{8}\frac{{{\text{d}}^{2} \hat{U}(\theta )}}{{{\text{d}}\theta^{2} }}} \right) + \frac{{\rho h\omega^{2} }}{\alpha }\hat{U}(\theta ) = 0, \hfill \\ \end{gathered} $$

14-2 $$ \begin{gathered} \frac{{{\text{d}}^{2} \hat{V}(\theta )}}{{{\text{d}}\theta^{2} }} + \frac{{{\text{d}}\hat{W}(\theta )}}{{{\text{d}}\theta }} - \frac{1}{2}\lambda_{m} (1 + \mu )\frac{{{\text{d}}\hat{U}(\theta )}}{{{\text{d}}\theta }} - \frac{1}{2}\lambda_{m}^{2} (1 - \mu )\hat{V}(\theta ) + \beta \left( {\frac{1}{2}\lambda_{m}^{2} (3 - \mu )\frac{{{\text{d}}\hat{W}(\theta )}}{{{\text{d}}\theta }}} \right. \hfill \\ \left. { \, - \frac{9}{8}\lambda_{m}^{2} (1 - \mu )\hat{V}(\theta ) + \frac{3}{8}\lambda_{m} (1 - \mu )\frac{{{\text{d}}\hat{U}(\theta )}}{{{\text{d}}\theta }} - \frac{{{\text{d}}^{3} \hat{W}(\theta )}}{{{\text{d}}\theta^{3} }} + \frac{{{\text{d}}^{2} \hat{V}(\theta )}}{{{\text{d}}\theta^{2} }}} \right) + \frac{{\rho h\omega^{2} }}{\alpha }\hat{V}(\theta ) = 0, \hfill \\ \end{gathered} $$

14-3 $$ \begin{gathered} \beta \left( { - \lambda_{m}^{4} \hat{W}(\theta ) + 2\lambda_{m}^{2} \frac{{{\text{d}}^{2} \hat{W}(\theta )}}{{{\text{d}}\theta^{2} }} - \frac{1}{2}\lambda_{m}^{2} (3 - \mu )\frac{{{\text{d}}\hat{V}(\theta )}}{{{\text{d}}\theta }}} \right. + \frac{1}{2}\lambda_{m} (1 - \mu )\frac{{{\text{d}}^{2} \hat{U}(\theta )}}{{{\text{d}}\theta^{2} }} \hfill \\ \, \left. { - \frac{{{\text{d}}^{4} \hat{W}(\theta )}}{{{\text{d}}\theta^{4} }} + \frac{{{\text{d}}^{3} \hat{V}(\theta )}}{{{\text{d}}\theta^{3} }}} \right) - \frac{{{\text{d}}\hat{V}(\theta )}}{{{\text{d}}\theta }} - \hat{W}(\theta ) + \lambda_{m} \mu \hat{U}(\theta ) + \frac{{\rho h\omega^{2} }}{\alpha }\hat{W}(\theta ) = 0, \hfill \\ \end{gathered} $$

where the non-dimensional parameter $\lambda_{m}$ belongs to the axial wave number and is defined by:

15 $$ \lambda_{m} = \frac{{mR{\uppi }}}{L} $$

As it is cleared from Eqs. (14-1)–(14-3), three ordinary differential equations (ODEs) of varying orders (2, 3, and 4, respectively) are obtained. To tackle such ODEs effectively, the DTM—a powerful technique introduced in the following section—is considered. In the subsequent section, DTM is applied to these equations, leveraging its efficacy in solving complex differential equations.

## Differential transform method (DTM)

In this section, by definition, the differential transform of the $k{\text{th}}$ derivative of the one variable continuously differentiable function $F(\xi )$ about the point $\xi = 0$ can be written as:

16 $$ \overline{F}[k] = \frac{1}{k!}\left[ {\frac{{{\text{d}}^{k} F(\xi )}}{{{\text{d}}\xi^{k} }}} \right]_{\xi = 0} , $$

where, $k$ is a non-negative integer number. In addition, herein and in what follows, a bar over a variable signifies the differential transform of the original function. On the other hand, the inverse differential transform which leads to the original function can be expressed as:

17 $$ F(\xi ) = \sum\limits_{k = 0}^{\infty } {\xi^{k} } \overline{F}[k]. $$

Finally, Substituting Eq. (16) into Eq. (17) results in:

18 $$ F(\xi ) = \sum\limits_{k = 0}^{\infty } {\frac{{\xi^{k} }}{k!}\left[ {\frac{{{\text{d}}^{k} F(\xi )}}{{{\text{d}}\xi^{k} }}} \right]_{\xi = 0} } , $$

where, the foregoing relation is the well-known Taylor series expansion of the function $F(\xi )$ about the point $\xi = 0$. It should be mentioned, for simplicity of calculation in practical purposes, Eq. (17) is converted into a finite series:

19 $$ F(\xi ) = \sum\limits_{k = 0}^{N} {\xi^{k} } \overline{F}[k], $$

in which, $N$ is the number of series terms determined by monitoring the convergence of the results. For the sake of completeness, some basic mathematical operations between original functions $F(\xi )$, $G(\xi )$ and $H(\xi )$ and their $k^{{{\text{th}}}}$ differential transform are listed in Table 1.

**Table 1:** Some fundamental operations of the DTM.

Original function	Transformed function
$F(\xi ) = G(\xi ) \pm H(\xi )$	$\overline{F}[k] = \overline{G}[k] \pm \overline{H}[k]$
$F(\xi ) = \gamma G(\xi ), \, \gamma \in R$	$\overline{F}[k] = \gamma \overline{G}[k]$
$F(\xi ) = \frac{{{\text{d}}G(\xi )}}{{{\text{d}}\xi }}$	$\overline{F}[k] = (k + 1)\overline{G}[k + 1]$
$F(\xi ) = \frac{{{\text{d}}^{n} G(\xi )}}{{{\text{d}}\xi^{n} }}$	$\overline{F}[k] = \frac{(k + n)!}{{n!}}\overline{G}[k + n]$
$F(\xi ) = G(\xi ) \, H(\xi )$	$\overline{F}[k] = \sum\nolimits_{l = 0}^{k} {\overline{G}[l] \, \overline{H}[k - l]}$
$F(\xi ) = \xi^{n}$	$$\overline{F}[k] = {\updelta }(k - n) = \left\{ {\begin{array}{*{20}c} 1 & {{\text{if }}k = n} \\ 0 & {{\text{if }}k \ne n} \\ \end{array} } \right.$$

## Applying DTM to free vibration problem

To simplify the calculations and align Eq. (14) more closely with the DTM, all parameters are expressed in dimensionless form. This is achieved by introducing the non-dimensional quantity $\xi$ as:

20 $$ \xi = \frac{{\theta - {\uppi }}}{{\uppi }} \, \Rightarrow \, \frac{\partial \xi }{{\partial \theta }} = \frac{1}{{\uppi }}, $$

in which, $\theta$ is the central angle of the closed cylinder as shown in Fig. 1a, and has a range between $0$ and $2{\uppi }$. As a result, $\xi$ varied between $- 1$ through $+ 1$. Subsequently, employing the chain rule results in:

21 $$ \frac{\partial }{\partial \theta } = \frac{\partial }{\partial \xi } \times \frac{\partial \xi }{{\partial \theta }} = \frac{1}{{\uppi }} \times \frac{\partial }{\partial \xi }. $$

Therefore, relations of Eq. (14) can be recast in the following form:

22-1 $$ \begin{gathered} \left( {\frac{1}{2} + \frac{1}{8}\beta } \right)(1 - \mu )\left( {\frac{1}{{\uppi }}} \right)^{2} U^{\prime\prime} + \left[ {\frac{{\rho h\omega^{2} }}{\alpha } - \lambda_{m}^{2} } \right]U + \left[ {\frac{1}{2}\lambda_{m} (1 + \mu ) - \frac{3}{8}\lambda_{m} \beta (1 - \mu )} \right]\left( {\frac{1}{{\uppi }}} \right)V^{\prime} \hfill \\ + \frac{1}{2}\lambda_{m} \beta (1 - \mu )\left( {\frac{1}{{\uppi }}} \right)^{2} W^{\prime\prime} + \lambda_{m} \mu W = 0, \hfill \\ \end{gathered} $$

22-2 $$ \begin{gathered} \left[ { - \frac{1}{2}\lambda_{m} (1 + \mu ) + \frac{3}{8}\lambda_{m} \beta (1 - \mu )} \right]\left( {\frac{1}{{\uppi }}} \right)U^{\prime} + (1 + \beta )\left( {\frac{1}{{\uppi }}} \right)^{2} V^{\prime\prime} + \left[ {\frac{{\rho h\omega^{2} }}{\alpha } - \lambda_{m}^{2} \left( {\frac{1}{2} + \frac{9}{8}\beta } \right)(1 - \mu )} \right]V \hfill \\ - \beta \left( {\frac{1}{{\uppi }}} \right)^{3} W^{\prime\prime} + \left[ {\frac{1}{2}\lambda_{m}^{2} \beta (3 - \mu ) + 1} \right]\left( {\frac{1}{{\uppi }}} \right)W^{\prime} = 0, \hfill \\ \end{gathered} $$

22-3 $$ \begin{gathered} \frac{1}{2}\lambda_{m} \beta (1 - \mu )\left( {\frac{1}{{\uppi }}} \right)^{2} U^{\prime\prime} + \lambda_{m} \mu U + \beta \left( {\frac{1}{{\uppi }}} \right)^{3} V^{\prime\prime\prime} - \left[ {\frac{1}{2}\lambda_{m}^{2} \beta (3 - \mu ) + 1} \right]\left( {\frac{1}{{\uppi }}} \right)V^{\prime} - \beta \left( {\frac{1}{{\uppi }}} \right)^{4} W^{\prime\prime\prime}{\prime} \hfill \\ + 2\lambda_{m}^{2} \beta \left( {\frac{1}{{\uppi }}} \right)^{2} W^{\prime\prime} + \left[ {\frac{{\rho h\omega^{2} }}{\alpha } - \lambda_{m}^{4} \beta - 1} \right]W = 0, \hfill \\ \end{gathered} $$

herein, the prime notation signifies the derivative with respect to $\xi$. In the following, by means of the foregoing definitions and the basic rules tabulated in Table 1, the governing differential equations of the shell, i.e. relations in Eq. (22), take the form of a set of recurrent equations as:

23-1 $$ \begin{gathered} \left( {\frac{1}{2} + \frac{1}{8}\beta } \right)(1 - \mu )\left( {\frac{1}{{\uppi }}} \right)^{2} (k + 1)(k + 2)\overline{U}[k + 2] + \left[ {\frac{{\rho h\omega^{2} }}{\alpha } - \lambda_{m}^{2} } \right]\overline{U}[k] \hfill \\ + \left[ {\frac{1}{2}\lambda_{m} (1 + \mu ) - \frac{3}{8}\lambda_{m} \beta (1 - \mu )} \right]\left( {\frac{1}{{\uppi }}} \right)(k + 1)\overline{V}[k + 1] \hfill \\ + \frac{1}{2}\left( {\frac{1}{{\uppi }}} \right)^{2} \lambda_{m} \beta (1 - \mu )(k + 1)(k + 2)\overline{W}[k + 2] + \lambda_{m} \mu \overline{W}[k] = 0, \hfill \\ \end{gathered} $$

23-2 $$ \begin{gathered} \left[ { - \frac{1}{2}\lambda_{m} (1 + \mu ) + \frac{3}{8}\lambda_{m} \beta (1 - \mu )} \right]\left( {\frac{1}{{\uppi }}} \right)(k + 1)\overline{U}[k + 1] + (1 + \beta )\left( {\frac{1}{{\uppi }}} \right)^{2} (k + 1)(k + 2)\overline{V}[k + 2] \hfill \\ + \left[ {\frac{{\rho h\omega^{2} }}{\alpha } - \lambda_{m}^{2} \left( {\frac{1}{2} + \frac{9}{8}\beta } \right)(1 - \mu )} \right]\overline{V}[k] - \beta \left( {\frac{1}{{\uppi }}} \right)^{3} (k + 1)(k + 2)(k + 3)\overline{W}[k + 3] \hfill \\ + 2\left( {\frac{1}{{\uppi }}} \right)^{2} \lambda_{m}^{2} \beta (k + 1)(k + 2)\overline{W}[k + 2] + \left[ {\frac{{\rho h\omega^{2} }}{\alpha } - \lambda_{m}^{4} \beta - 1} \right]\overline{W}[k] = 0, \hfill \\ \end{gathered} $$

23-3 $$ \begin{gathered} \frac{1}{2}\left( {\frac{1}{{\uppi }}} \right)^{2} \lambda_{m} \beta (1 - \mu )(k + 1)(k + 2)\overline{U}[k + 2] + \lambda_{m} \mu \overline{U}[k] + \beta \left( {\frac{1}{{\uppi }}} \right)^{3} (k + 1)(k + 2)(k + 3)\overline{V}[k + 3] \hfill \\ - \left[ {\frac{1}{2}\lambda_{m}^{2} \beta (3 - \mu ) + 1} \right]\left( {\frac{1}{{\uppi }}} \right)(k + 1)\overline{V}[k + 1] - \beta \left( {\frac{1}{{\uppi }}} \right)^{4} (k + 1)(k + 2)(k + 3)(k + 4)\overline{W}[k + 4] \hfill \\ + 2\left( {\frac{1}{{\uppi }}} \right)^{2} \lambda_{m}^{2} \beta (k + 1)(k + 2)\overline{W}[k + 2] + \left[ {\frac{{\rho h\omega^{2} }}{\alpha } - \lambda_{m}^{4} \beta - 1} \right]\overline{W}[k] = 0, \hfill \\ \end{gathered} $$

where, $\overline{U}[k]$, $\overline{V}[k]$ and $\overline{W}[k]$ are the $k^{{{\text{th}}}}$ differential transform of the unknown functions $U(\xi )$, $V(\xi )$ and $W(\xi )$,correspondingly. In this context, the method for changing the variables and marking them for the reader's convenience is presented in Eq. (24). From that, it is evident that the notations differ, and the main equations to distinguish them are Eqs. (6), (10), (14), (22), and (23), respectively. The following relation shows the notation changes for the first unknown variable, as an example:

24 $$ \begin{gathered} u(x,\theta ,t)\,\mathop{\longrightarrow}\limits^{{\text{Fourier transform}}}\,u^{*} (x,\theta ,\omega )\,\mathop{\longrightarrow}\limits^{{\sin \frac{m\pi x}{L}}}\,\hat{U}(\theta ,\omega ) \\ \,\mathop{\longrightarrow}\limits^{{{\text{non}} - {\text{dimensional}}}}\,U(\xi ,\omega )\,\mathop{\longrightarrow}\limits^{{{\text{DTM}}}}\,\overline{U} (k,\omega ) \\ \end{gathered} $$

It seems apparent that the recurrence relations in Eq. (23) are still coupled and elaborate. Now, by solving the set of preceding equations simultaneously, the following recursive formulas can be achieved:

25-1 $$ \begin{gathered} \overline{U}[k + 2] = - \frac{8}{(\beta + 4)(1 - \mu )(k + 1)(k + 2)}\left\{ {\left[ {\frac{{\rho h\omega^{2} }}{\alpha } - \lambda_{m}^{2} } \right]{\uppi }^{2} \overline{U}[k]} \right. \hfill \\ \, + \left[ {\frac{1}{2}\lambda_{m} (1 + \mu ) - \frac{3}{8}\lambda_{m} \beta (1 - \mu )} \right]{\uppi }(k + 1)\overline{V}[k + 1] \hfill \\ \, \left. { + \frac{1}{2}\lambda_{m} \beta (1 - \mu )(k + 1)(k + 2)\overline{W}[k + 2] + \lambda_{m} \mu {\uppi }^{2} \overline{W}[k]} \right\}, \hfill \\ \end{gathered} $$

25-2 $$ \begin{gathered} \overline{V}[k + 2] = \frac{1}{{{\uppi }(1 + \beta )(k + 1)(k + 2)}}\left\{ {\left[ {\frac{1}{2}\lambda_{m} (1 + \mu ) - \frac{3}{8}\lambda_{m} \beta (1 - \mu )} \right]{\uppi }^{2} (k + 1)\overline{U}[k + 1]} \right. \hfill \\ \, - \left[ {\frac{{\rho h\omega^{2} }}{\alpha } - \lambda_{m}^{2} (\frac{1}{2} + \frac{9}{8}\beta )(1 - \mu )} \right]{\uppi }^{3} \overline{V}[k] + \beta (k + 1)(k + 2)(k + 3)\overline{W}[k + 3] \hfill \\ \, \left. { - \left[ {\frac{1}{2}\lambda_{m}^{2} \beta (3 - \mu ) + 1} \right]{\uppi }^{2} (k + 1)\overline{W}[k + 1]} \right\}, \hfill \\ \end{gathered} $$

25-3 $$ \begin{gathered} \overline{W}[k + 4] = \frac{1}{\beta (k + 1)(k + 2)(k + 3)(k + 4)}\left\{ {\frac{1}{2}\lambda_{m} \beta {\uppi }^{2} (1 - \mu )(k + 1)(k + 2)\overline{U}[k + 2] + \lambda_{m} \mu {\uppi }^{4} \overline{U}[k]} \right. \hfill \\ \, + \beta {\uppi }(k + 1)(k + 2)(k + 3)\overline{V}[k + 3] - \left[ {\frac{1}{2}\lambda_{m}^{2} \beta (3 - \mu ) + 1} \right]{\uppi }^{3} (k + 1)\overline{V}[k + 1] \hfill \\ \, \left. { + 2\lambda_{m}^{2} \beta {\uppi }^{2} (k + 1)(k + 2)\overline{W}[k + 2] + \left[ {\frac{{\rho h\omega^{2} }}{\alpha } - \lambda_{m}^{4} \beta - 1} \right]{\uppi }^{4} \overline{W}[k]} \right\}. \hfill \\ \end{gathered} $$

It is clearly visible that by employing the foregoing relations in Eq. (25), one can produce sufficient terms of recurrence expression $\overline{U}[k]$, $\overline{V}[k]$ and $\overline{W}[k]$. For a better comprehension of the proposed procedure, a few terms of the series were calculated in detail. To this end, first, the initial values for $\overline{U}[k]$, $\overline{V}[k]$ and $\overline{W}[k]$ are assumed as:

26 $$ \begin{array}{*{20}c} {\overline{U}[0] = C_{1} ,} & {\overline{U}[1] = C_{2} ,} & {} & {} \\ {\overline{V}[0] = C_{3} ,} & {\overline{V}[1] = C_{4} ,} & {} & {} \\ {\overline{W}[0] = C_{5} ,} & {\overline{W}[1] = C_{6} ,} & {\overline{W}[2] = C_{7} ,} & {\overline{W}[3] = C_{8} .} \\ \end{array} $$

Therefore, by setting $k = 0$ in the recursive formulas one can obtain:

27-1 $$ \begin{gathered} \overline{U}[2] = - \frac{4}{(\beta + 4)(1 - \mu )}\left\{ {\left[ {\frac{{\rho h\omega^{2} }}{\alpha } - \lambda_{m}^{2} } \right]{\uppi }^{2} C_{1} } \right. + \left[ {\frac{1}{2}\lambda_{m} (1 + \mu ) - \frac{3}{8}\lambda_{m} \beta (1 - \mu )} \right]{\uppi }C_{4} \hfill \\ \, \left. { + \lambda_{m} \beta (1 - \mu )C_{7} + \lambda_{m} \mu {\uppi }^{2} C_{5} } \right\}, \hfill \\ \end{gathered} $$

27-2 $$ \begin{gathered} \overline{V}[2] = \frac{1}{2\pi (1 + \beta )}\left\{ {\left[ {\frac{1}{2}\lambda_{m} (1 + \mu ) - \frac{3}{8}\lambda_{m} \beta (1 - \mu )} \right]\pi^{2} C_{2} } \right. - \left[ {\frac{{\rho h\omega^{2} }}{\alpha } - \lambda_{m}^{2} \left( {\frac{1}{2} + \frac{9}{8}\beta } \right)(1 - \mu )} \right]\pi^{3} C_{3} \hfill \\ \left. { + 6\beta C_{8} - \left[ {\frac{1}{2}\lambda_{m}^{2} \beta (3 - \mu ) + 1} \right]\pi^{2} C_{6} } \right\}. \hfill \\ \end{gathered} $$

On the other hand, setting $k = 0$ in Eq. (25-3) to obtain $\overline{W}[4]$ requires the value of $\overline{V}[3]$ which is still not calculated. Notwithstanding this, setting $k = 1$ in Eq. (25-2) obliges to have the unknown parameter $\overline{W}[4][4]$. Consequently, the Eqs. (25-2) and (25–3) are still coupled. To address this difficulty, the authors proposed to solve the last two relations of Eq. (25) simultaneously. For this purpose, first, $k$ in Eq. (25-2) is substituted by $k + 1$ as:

28 $$ \begin{gathered} \overline{V}[k + 3] = \frac{1}{{{\uppi }(1 + \beta )(k + 2)(k + 3)}}\left\{ {\left[ {\frac{1}{2}\lambda_{m} (1 + \mu ) - \frac{3}{8}\lambda_{m} \beta (1 - \mu )} \right]{\uppi }^{2} (k + 2)\overline{U}[k + 2]} \right. \hfill \\ \, - \left[ {\frac{{\rho h\omega^{2} }}{\alpha } - \lambda_{m}^{2} (\frac{1}{2} + \frac{9}{8}\beta )(1 - \mu )} \right]{\uppi }^{3} \overline{V}[k + 1] + \beta (k + 2)(k + 3)(k + 4)\overline{W}[k + 4] \hfill \\ \, \left. { - \left[ {\frac{1}{2}\lambda_{m}^{2} \beta (3 - \mu ) + 1} \right]{\uppi }^{2} (k + 2)\overline{W}[k + 2]} \right\}. \hfill \\ \end{gathered} $$

In the next stage, Eqs. (25–3) and (28) can be recast in the following form:

29-1 $$ \begin{gathered} \beta (k + 1)(k + 2)(k + 3)(k + 4)\overline{W}[k + 4] - \beta {\uppi }(k + 1)(k + 2)(k + 3)\overline{V}[k + 3] \hfill \\ = \frac{1}{2}\lambda_{m} \beta {\uppi }^{2} (1 - \mu )(k + 1)(k + 2)\overline{U}[k + 2] + \lambda_{m} \mu {\uppi }^{4} \overline{U}[k] \hfill \\ - \left[ {\frac{1}{2}\lambda_{m}^{2} \beta (3 - \mu ) + 1} \right]{\uppi }^{3} (k + 1)\overline{V}[k + 1] + 2\lambda_{m}^{2} \beta {\uppi }^{2} (k + 1)(k + 2)\overline{W}[k + 2] \hfill \\ \, + \left[ {\frac{{\rho h\omega^{2} }}{\alpha } - \lambda_{m}^{4} \beta - 1} \right]{\uppi }^{4} \overline{W}[k], \hfill \\ \end{gathered} $$

29-2 $$ \begin{gathered} \beta (k + 2)(k + 3)(k + 4)\overline{W}[k + 4] - {\uppi }(1 + \beta )(k + 2)(k + 3)\overline{V}[k + 3] \hfill \\ = - \left[ {\frac{1}{2}\lambda_{m} (1 + \mu ) - \frac{3}{8}\lambda_{m} \beta (1 - \mu )} \right]{\uppi }^{2} (k + 2)\overline{U}[k + 2] \hfill \\ + \left[ {\frac{{\rho h\omega^{2} }}{\alpha } - \lambda_{m}^{2} \left( {\frac{1}{2} + \frac{9}{8}\beta } \right)(1 - \mu )} \right]{\uppi }^{3} \overline{V}[k + 1] + \left[ {\frac{1}{2}\lambda_{m}^{2} \beta (3 - \mu ) + 1} \right]{\uppi }^{2} (k + 2)\overline{W}[k + 2]. \hfill \\ \end{gathered} $$

Over the following step, solving the set of Eq. (29) give rise to:

30-1 $$ \begin{gathered} \overline{V}[k + 3] = \frac{1}{(k + 1)(k + 2)(k + 3)}\left\{ {\left[ {\frac{1}{2}\lambda_{m} (1 + \mu ) + \frac{1}{8}\lambda_{m} \beta (1 - \mu )} \right]{\uppi }(k + 1)(k + 2)\overline{U}[k + 2]} \right. \hfill \\ + \lambda_{m} \mu {\uppi }^{3} \overline{U}[k] - \left[ {\frac{{\rho h\omega^{2} }}{\alpha } - \frac{1}{2}\lambda_{m}^{2} \left[ {(1 - \mu ) - \beta \left( {\frac{3}{4} + \frac{5}{4}\mu } \right)} \right] + 1} \right]{\uppi }^{2} (k + 1)\overline{V}[k + 1] \hfill \\ \left. { + \left[ {\frac{1}{2}\lambda_{m}^{2} \beta (1 + \mu ) - 1} \right]{\uppi }(k + 1)(k + 2)\overline{W}[k + 2] + \left[ {\frac{{\rho h\omega^{2} }}{\alpha } - \lambda_{m}^{4} \beta - 1} \right]{\uppi }^{3} \overline{W}[k]} \right\}, \hfill \\ \end{gathered} $$

30-2 $$ \begin{gathered} \overline{W}[k + 4] = \frac{1}{(k + 1)(k + 2)(k + 3)(k + 4)}\left\{ {\frac{1}{8}\left[ {8 + \beta (1 - \mu )} \right]\lambda_{m} {\uppi }^{2} (k + 1)(k + 2)\overline{U}[k + 2]} \right. \hfill \\ + \lambda_{m} \mu \left( {1 + \frac{1}{\beta }} \right){\uppi }^{4} \overline{U}[k] - \left[ {\frac{{\rho h\omega^{2} }}{\alpha } + \frac{1}{8}\lambda_{m}^{2} [8 + \beta (3 + 5\mu )] + \left( {1 + \frac{1}{\beta }} \right)} \right]{\uppi }^{3} (k + 1)\overline{V}[k + 1] \hfill \\ + \left[ {\frac{1}{2}\lambda_{m}^{2} [4 + \beta (1 + \mu )] - 1} \right]{\uppi }^{2} (k + 1)(k + 2)\overline{W}[k + 2] \hfill \\ \left. { + \left[ {\frac{{\rho h\omega^{2} }}{\alpha } - \lambda_{m}^{4} \beta - 1} \right]\left( {1 + \frac{1}{\beta }} \right){\uppi }^{4} \overline{W}[k]} \right\}. \hfill \\ \end{gathered} $$

It is pertinent to recall that the parameters $\alpha$, $\beta$ and $\lambda_{m}$ are defined as follows:

$$ \begin{gathered} \alpha = \frac{Eh}{{R^{2} (1 - \mu^{2} )}},\,\,\,\,{\text{Rep}}{.}\,\,(7 - 1) \hfill \\ \beta = \frac{{h^{2} }}{{12R^{2} }},\,\,\,\,\,\,\,\,\,\,\,\,\,\,\,{\text{Rep}}{.}\,\,(7 - 2) \hfill \\ \lambda_{m} = \frac{{mR{\uppi }}}{L}.\,\,\,\,\,\,\,\,\,\,\,\,\,\,{\text{Rep}}{.}\,\,(15) \hfill \\ \end{gathered} $$

Consequently, setting $k = 0$ in the relations of Eq. (30) leads to:

31-1 $$ \begin{gathered} \overline{V}[3] = \frac{1}{6}\left\{ {\left[ {\lambda_{m} (1 + \mu ) + \frac{1}{4}\lambda_{m} \beta (1 - \mu )} \right]{\uppi }\overline{U}[2] + \lambda_{m} \mu {\uppi }^{3} C_{1} } \right. \hfill \\ - \left[ {\frac{{\rho h\omega^{2} }}{\alpha } - \frac{1}{2}\lambda_{m}^{2} \left[ {(1 - \mu ) - \beta \left( {\frac{3}{4} + \frac{5}{4}\mu } \right)} \right] + 1} \right]{\uppi }^{2} C_{4} + 2{\uppi }\left[ {\frac{1}{2}\lambda_{m}^{2} \beta (1 + \mu ) - 1} \right]C_{7} \hfill \\ \left. { + \left[ {\frac{{\rho h\omega^{2} }}{\alpha } - \lambda_{m}^{4} \beta - 1} \right]{\uppi }^{3} C_{5} } \right\}, \hfill \\ \end{gathered} $$

31-2 $$ \begin{gathered} \overline{W}[4] = \frac{1}{24}\left\{ {\frac{1}{4}\left[ {8 + \beta (1 - \mu )} \right]\lambda_{m} \pi^{2} \overline{U}[2] + \lambda_{m} \mu \left( {1 + \frac{1}{\beta }} \right)\pi^{4} C_{1} } \right. \hfill \\ - \left[ {\frac{{\rho h\omega^{2} }}{\alpha } + \frac{1}{8}\lambda_{m}^{2} [8 + \beta (3 + 5\mu )] + \left( {1 + \frac{1}{\beta }} \right)} \right]\pi^{3} C_{4} + 2\pi^{2} \left[ {\frac{1}{2}\lambda_{m}^{2} [4 + \beta (1 + \mu )] - 1} \right]C_{7} \hfill \\ \left. { + \left[ {\frac{{\rho h\omega^{2} }}{\alpha } - \lambda_{m}^{4} \beta - 1} \right]\left( {1 + \frac{1}{\beta }} \right)\pi^{4} C_{5} } \right\}. \hfill \\ \end{gathered} $$

It should be emphasized $\overline{U}[2]$ in the preceding relations were obtained previously in Eq. (27-1). In a same manner, one can obtain $\overline{U}[3]$, $\overline{V}[4]$ and $\overline{W}[5]$ by setting $k = 1$ in the Eqs. (25-1), (30-1) and (31-2), correspondingly. Likewise, using $k = 2$ in the mentioned relations results in $\overline{U}[4]$, $\overline{V}[5]$ and $\overline{W}[6]$, respectively. The same procedure can be continued until sufficient terms of the parameters of recurrence expression $\overline{U}[k]$, $\overline{V}[k]$ and $\overline{W}[k]$ are produced. Finally, with the aid of Eq. (19), the unknown functions $U(\xi )$, $V(\xi )$ and $W(\xi )$ can be computed as:

32-1 $$ U(\xi ) = \sum\limits_{k = 0}^{N + 2} {\xi^{k} } \overline{U}[k], $$

32-2 $$ V(\xi ) = \sum\limits_{k = 0}^{N + 3} {\xi^{k} } \overline{V}[k], $$

32-3 $$ W(\xi ) = \sum\limits_{k = 0}^{N + 4} {\xi^{k} } \overline{W}[k]. $$

### Differential transformation of boundary and compatibility equations

As discussed in the previous section, the main aim of the present paper is to study the closed circular cylinders with freely simply supported at both ends. It is beneficial to remember that the following conditions are applied along the longitudinal direction of the cylinders:

$$ N_{xx} = M_{xx} = v = w = 0\,\,\,\,\,{\text{at}}\,\,\,\,\,\,x = 0,L.\,\,\, {\text{Rep}}.\left( {{12}} \right) $$

Besides, in the circumferential direction, certain periodicity requirements must be used instead of the boundary conditions as:

33 $$ \begin{array}{*{20}l} { \bullet \, u(x,0,t) = u(x,2{\pi ,}t)} \hfill & { \bullet \, T_{x\theta } (x,0,t) = T_{x\theta } (x,2{\pi ,}t)} \hfill \\ { \bullet \, v(x,0,t) = v(x,2{\pi ,}t)} \hfill & { \bullet \, N_{\theta \theta } (x,0,t) = N_{\theta \theta } (x,2{\pi ,}t)} \hfill \\ { \bullet \, w(x,0,t) = w(x,2{\pi ,}t)} \hfill & { \bullet \, V_{\theta } (x,0,t) = V_{\theta } (x,2{\pi ,}t)} \hfill \\ {\left. { \bullet \, \frac{\partial w(x,\theta ,t)}{{\partial \theta }}} \right|_{\theta = 0} = \left. {\frac{\partial w(x,\theta ,t)}{{\partial \theta }}} \right|_{{\theta = 2{\uppi }}} } \hfill & { \bullet \, M_{\theta \theta } (x,0,t) = M_{\theta \theta } (x,2{\pi ,}t)} \hfill \\ \end{array} $$

in which, the effective transverse shear force, $V_{\theta }$, and $T_{x\theta }$ are defined as:

34 $$ \begin{gathered} V_{\theta } = Q_{\theta } + \frac{{\partial M_{x\theta } }}{\partial x} = \frac{1}{R}\frac{{\partial M_{\theta \theta } }}{\partial \theta } + 2\frac{{\partial M_{x\theta } }}{\partial x}, \hfill \\ T_{x\theta } = N_{x\theta } - \frac{{M_{x\theta } }}{2R}. \hfill \\ \end{gathered} $$

On the other hand, substituting Eqs. (3) and (4) in the foregoing relations lead to:

35 $$ \begin{gathered} T_{x\theta } = D(1 - \mu )\left[ {\frac{1}{2}\frac{\partial v}{{\partial x}} + \frac{1}{2R}\frac{\partial u}{{\partial \theta }}} \right] - \eta (1 - \mu )\left[ { - \frac{1}{{2R^{2} }}\frac{{\partial^{2} w}}{\partial x\partial \theta } + \frac{3}{{8R^{2} }}\frac{\partial v}{{\partial x}} - \frac{1}{{8R^{3} }}\frac{\partial u}{{\partial \theta }}} \right], \hfill \\ V_{\theta } = \eta \left[ {\left( { - \frac{1}{{R^{3} }}\frac{{\partial^{3} w}}{{\partial \theta^{3} }} + \frac{1}{{R^{3} }}\frac{{\partial^{2} v}}{{\partial \theta^{2} }}} \right) + \mu \left( { - \frac{1}{R}\frac{{\partial^{3} w}}{{\partial x^{2} \partial \theta }}} \right)} \right] + 2\eta (1 - \mu )\left[ { - \frac{1}{R}\frac{{\partial^{3} w}}{{\partial x^{2} \partial \theta }} + \frac{3}{4R}\frac{{\partial^{2} v}}{{\partial x^{2} }} - \frac{1}{{4R^{2} }}\frac{{\partial^{2} u}}{\partial x\partial \theta }} \right], \hfill \\ M_{\theta \theta } = \eta \left[ {\left( { - \frac{1}{{R^{2} }}\frac{{\partial^{2} w}}{{\partial \theta^{2} }} + \frac{1}{{R^{2} }}\frac{\partial v}{{\partial \theta }}} \right) + \mu \left( { - \frac{{\partial^{2} w}}{{\partial x^{2} }}} \right)} \right], \hfill \\ N_{\theta \theta } = D\left[ {\left( {\frac{1}{R}\frac{\partial v}{{\partial \theta }} + \frac{w}{R}} \right) + \mu \left( {\frac{\partial u}{{\partial x}}} \right)} \right]. \hfill \\ \end{gathered} $$

It should be pointed out that the preceding compatibility conditions, after some algebraic operations, could be simplified as:

36 $$ \begin{array}{*{20}l} { \bullet \, u(x,0,t) = u(x,2{\pi ,}t)} \hfill & { \bullet \, u^{\prime}(x,0,t) = u^{\prime}(x,2{\pi ,}t)} \hfill \\ { \bullet \, v(x,0,t) = v(x,2{\pi ,}t)} \hfill & { \bullet \, v^{\prime}(x,0,t) = v^{\prime}(x,2{\pi ,}t)} \hfill \\ { \bullet \, w(x,0,t) = w(x,2{\pi ,}t)} \hfill & { \bullet \, V_{\theta } (x,0,t) = V_{\theta } (x,2{\pi ,}t)} \hfill \\ {\left. { \bullet \, \frac{\partial w(x,\theta ,t)}{{\partial \theta }}} \right|_{\theta = 0} = \left. {\frac{\partial w(x,\theta ,t)}{{\partial \theta }}} \right|_{{\theta = 2{\uppi }}} } \hfill & { \bullet \, \left. {\frac{{\partial^{2} w(x,\theta ,t)}}{{\partial \theta^{2} }}} \right|_{\theta = 0} = \left. {\frac{{\partial^{2} w(x,\theta ,t)}}{{\partial \theta^{2} }}} \right|_{{\theta = 2{\uppi }}} } \hfill \\ \end{array} $$

In this research, two noteworthy aspects merit mention. Firstly, the boundary conditions outlined in Eq. (12) at the beginning and end of the cylindrical shell are designed to emulate the conditions of a cylindrical shell at both ends. Secondly, the boundary conditions described in Eq. (33) guarantees displacement and its derivatives continuity, involve $u(x,\theta ,t)$, $v(x,\theta ,t)$, and $w(x,\theta ,t)$ component. Additionally, $T_{x\theta } (x,\theta ,t)$, $N_{\theta \theta } (x,\theta ,t)$, and $V_{\theta } (x,\theta ,t)$ are defined to ensure force continuity at the connection point, while $\frac{\partial w(x,\theta ,t)}{{\partial \theta }}$ and $M_{\theta \theta } (x,\theta ,t)$ ensure continuity of slope and moment on both sides, respectively. In the next stage, the differential transform of the 8 periodicity requirements must be calculated. They have the following dimensionless shape:

37-1 $$ \sum\limits_{k = 0}^{N + 2} {( - 1)^{k} } \overline{U}[k] - \sum\limits_{k = 0}^{N + 2} {(1)^{k} } \overline{U}[k] = 0, $$

37-2 $$ \sum\limits_{k = 0}^{N + 1} {(k + 1)( - 1)^{k} } \overline{U}[k + 1] - \sum\limits_{k = 0}^{N + 1} {(k + 1)(1)^{k} } \overline{U}[k + 1] = 0, $$

37-3 $$ \sum\limits_{k = 0}^{N + 3} {( - 1)^{k} } \overline{V}[k] - \sum\limits_{k = 0}^{N + 3} {(1)^{k} } \overline{V}[k] = 0, $$

37-4 $$ \sum\limits_{k = 0}^{N + 2} {(k + 1)( - 1)^{k} } \overline{V}[k + 1] - \sum\limits_{k = 0}^{N + 2} {(k + 1)(1)^{k} } \overline{V}[k + 1] = 0, $$

37-5 $$ \sum\limits_{k = 0}^{N + 4} {( - 1)^{k} } \overline{W}[k] - \sum\limits_{k = 0}^{N + 4} {(1)^{k} } \overline{W}[k] = 0, $$

37-6 $$ \sum\limits_{k = 0}^{N + 3} {(k + 1)( - 1)^{k} } \overline{W}[k + 1] - \sum\limits_{k = 0}^{N + 3} {(k + 1)(1)^{k} } \overline{W}[k + 1] = 0, $$

37-7 $$ \sum\limits_{k = 0}^{N + 2} {(k + 1)(k + 2)( - 1)^{k} } \overline{W}[k + 2] - \sum\limits_{k = 0}^{N + 2} {(k + 1)(k + 2)(1)^{k} } \overline{W}[k + 2] = 0, $$

37-8 $$ \begin{gathered} - \frac{1}{{R^{3} }}(\frac{1}{{\uppi }})^{3} \left[ {\sum\limits_{k = 0}^{N + 1} {(k + 1)(k + 2)(k + 3)( - 1)^{k} } \overline{W}[k + 3] - \sum\limits_{k = 0}^{N + 1} {(k + 1)(k + 2)(k + 3)(1)^{k} } \overline{W}[k + 3]} \right] \hfill \\ \, + \frac{1}{{R^{3} }}\left( {\frac{1}{{\uppi }}} \right)^{2} \left[ {\sum\limits_{k = 0}^{N + 1} {(k + 1)(k + 2)( - 1)^{k} } \overline{V}[k + 2] - \sum\limits_{k = 0}^{N + 1} {(k + 1)(k + 2)(1)^{k} } \overline{V}[k + 2]} \right] = 0. \hfill \\ \end{gathered} $$

It is noteworthy to remember that by successive utilizing the relations (25-1) and (30) with the initial values mentioned in Eq. (26), one can produce $\overline{U}[k]$, $\overline{V}[k]$ and $\overline{W}[k]$ up to a sufficient number of terms. Afterwards, substitution of these generated recurrence relations into the preceding compatibility conditions leads to a set of homogenous equations which can rewritten in the next form:

38 $$ \left[ A \right]\left\{ C \right\} = \left\{ 0 \right\}, $$

in which, $[A]$ is the coefficient matrix and $\{ C\}$ is the vector of initial values of $\overline{U}[k]$, $\overline{V}[k]$ and $\overline{W}[k]$ and is defined as:

39 $$ \left\{ C \right\} = \left\{ {\begin{array}{*{20}c} {C_{1} } & {C_{2} } & {C_{3} } & {C_{4} } & {C_{5} } & {C_{6} } & {C_{7} } & {C_{8} } \\ \end{array} } \right\}^{{\text{T}}} . $$

Finally, equating the determinant of the coefficient matrix to zero gives the natural frequencies of the cylinder.

To ensure the precision of the DTM analysis, a hybrid finite element method was implemented, corroborating all outcomes derived from the DTM. This hybrid method employed Sander’s displacement functions, replacing standard algebraic polynomials, and featured specific panel elements. Strips with two linear nodes were utilized, each node encompassing four degrees of freedom (including three axial, radial, and peripheral degrees of freedom, along with one rotational degree of freedom) (38). The theoretical aspects of the problem were elucidated in this context. Subsequently, the following section delves into the analysis and comparison of the results obtained from the DTM and this hybrid method.

## Calculation and discussion

**Figure 2:**
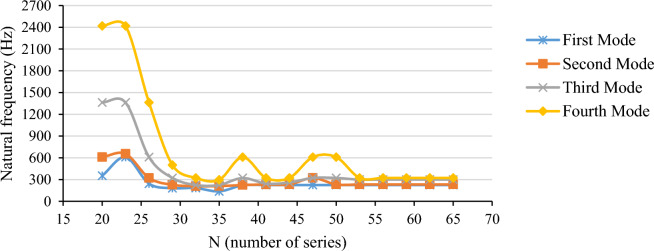
Convergence test for first forth modes.

A computer program was developed in-house to obtain the necessary results based on the mentioned solution method. This section thoroughly examines and discusses various findings. Following this, a detailed test is conducted to ensure the accuracy of the results, specifically focusing on the first four modes. The obtained results are compared to existing literature for validation. Additionally, comparisons are made between the first five modes' outcomes, including natural frequencies and mode shapes, and those obtained from the hybrid finite elements. Moreover, numerical computations are conducted to showcase the natural frequency curves concerning mechanical and geometric properties. The accuracy of the method is further verified through a convergence test, ensuring reliable outcomes. Figure 2 illustrates the relevant diagram, while Table 2 outlines the geometric and mechanical properties of the shell under analysis. To further clarify how the exact number of series terms was determined, Table 3 has also been provided.

**Table 2:** Mechanical properties of the shell.

Parameter	$E\,({\text{Pa}})$	$\upsilon$	$\rho \,\,({\text{kg}}/{\text{m}}^{3} )$	$t\,(m)$	$R\,(m)$	$L\,(m)$
Value	$206 \times 10^{9}$	$0.3$	$7680$	$0.001$	$0.175$	$0.664$

**Table 3:** Convergence test results and Errors.

N	1st mode		2nd mode		3rd mode		4th mode	
	Value	Error %	Value	Error %	Value	Error %	Value	Error %
30	180.8250	− 19.43	227.3687	− 2.07	321.8544	7.53	500.3559	55.45
32	182.9887	− 18.46	208.4900	− 10.20	223.9644	− 25.17	321.8669	0.00
35	136.2144	− 39.30	209.1386	− 9.92	224.4045	− 25.02	295.7667	− 8.10
38	220.1247	− 1.92	224.4276	− 3.33	321.8664	7.53	609.6414	89.40
40	224.4301	− 0.001	231.5173	− 0.28	244.2308	− 18.40	321.8664	0.00
43	224.4342	0.00	232.6123	0.18	258.8739	− 13.50	321.8664	0.00
47	224.4343	0.00	321.8664	38.62	321.8664	7.53	609.6414	89.40
50	224.4343	0.00	232.1789	0.00	321.8664	7.53	609.6414	89.40
52	224.4343	0.00	232.1796	0.00	298.7642	− 0.18	321.8664	0.00
55	224.4343	0.00	232.1795	0.00	299.2933	0.00	321.8664	0.00
58	224.4343	0.00	232.1795	0.00	299.2526	− 0.01	321.8664	0.00
62	224.4343	0.00	232.1795	0.00	299.3060	0.00	321.8664	0.00
65	224.4343	0.00	232.1795	0.00	299.3060	0.00	321.8664	0.00

The errors are calculated using the formula $Error = \frac{{Value(N_{65} ) - Value(N_{purposed\,\,row} )}}{{Value(N_{65} )}} \times 100$.

After confirming the method's final outcomes and the required series terms, their values are cross-verified with available data in published sources. This section presents a comparison between the data, featuring properties outlined in Table 2, and outputs derived from various studies in the literature, including Amabili ^5^, Lakis et al. ^33^, and Kashani et al. ^15^. Furthermore, a comparative analysis with the results obtained from the coded hybrid finite elements, specifically focusing on the first three modes, is presented and summarized in Table 4.

**Table 4:** Validation of natural frequencies (Hz) of cylindrical shells.

*n* (mode number)	Lakis et al. ^33^	Amabili (experimental) ^5^	Kashani and Aftabi ^15^	DTM	FEM	Error %
1	224.60	228.79	224.45	224.43	224.447	1.90
2	232.30	238.80	232.19	232.18	232.194	2.77
3	299.60	306.75	299.34	299.30	299.337	2.42

The errors are calculated using the formula ${\text{Error}} = \frac{{{\text{Amabilis}}\,{\text{Data}} - {\text{DTM}}}}{{{\text{Amabilis}}\,{\text{Data}}}} \times 100$.

Table 4 illustrates the remarkably high accuracy of the method's data, exhibiting negligible error percentages in comparison to other numerical methods.

In the following section and the main part of the research, the results of the DTM are presented. In the modeling, the mechanical and geometric properties which are presented in Table 2, are utilized. Moreover, in the analysis the finite element method was coded. As the following shows, the two methods are in good agreement with each other, indicating that the accuracy of DTM, as well as its simplicity. The first four modes of the cylindrical shell were evaluated in term of frequency and mode analysis and are shown in Table 5. It should be noted that the end support of the shell was free-simple ($V = W = 0$).

**Table 5:** Outcomes frequencies of closed cylindrical shell with DTM method and its comparison with FEM method.

Mode number	*n* = 1	*n* = 2	*n* = 3	*n* = 4
DTM	224.43	232.18	299.30	321.86
FEM	224.447	232.194	299.337	321.902
Error %	0.00	0.00	0.01	0.01

The errors are calculated using the formula ${\text{Error}} = \frac{{{\text{FEM}} - {\text{DTM}}}}{{{\text{FEM}}}} \times 100$.

In order to better compare the results of the mode shapes and identify any potential differences or inconsistencies, the two-dimensional mode shape was examined more precisely through a point-to-point comparison in Table 6 and Fig. 5.

**Table 6:** A comparison between the results of DTM’s and FEM’s 2D mode shapes.

Node	1st mode			2nd mode			3rd mode		
	DTM	FEM	Error %	DTM	FEM	Error %	DTM	FEM	Error %
1	0.1484	0.1470	− 0.90	0.2039	0.2037	− 0.06	0.1750	0.1766	0.94
2	0.1777	0.1767	− 0.57	0.1605	0.1553	− 3.36	0.1609	0.1580	− 1.79
3	0.2010	0.2025	0.76	0.1605	0.1659	3.23	0.1978	0.2007	1.42
4	0.1667	0.1674	0.41	0.2039	0.2037	− 0.06	0.1522	0.1502	− 1.27
5	0.1507	0.1490	− 1.13	0.1605	0.1553	− 3.36	0.1889	0.1892	0.13
6	0.1882	0.1879	− 0.18	0.1605	0.1659	3.23	0.1750	0.1766	0.93
7	0.1965	0.1982	0.89	0.2039	0.2037	− 0.06	0.1609	0.1580	− 1.83
8	0.1572	0.1571	− 0.01	0.1605	0.1553	− 3.36	0.1978	0.2007	1.44
9	0.1572	0.1554	− 1.13	0.1605	0.1659	3.23	0.1521	0.1502	− 1.24
10	0.1965	0.1969	0.19	0.2039	0.2037	− 0.06	0.1890	0.1892	0.10
11	0.1882	0.1899	0.88	0.1605	0.1553	− 3.36	0.1749	0.1766	0.95
12	0.1507	0.1499	− 0.49	0.1605	0.1659	3.23	0.1610	0.1580	− 1.87
13	0.1667	0.1652	− 0.92	0.2039	0.2037	− 0.06	0.1977	0.2007	1.46
14	0.2010	0.2020	0.52	0.1605	0.1553	− 3.36	0.1521	0.1502	− 1.22
15	0.1777	0.1790	0.72	0.1605	0.1659	3.23	0.1890	0.1892	0.07
	Average of absolute value of errors		0.65	Average of absolute value of errors		2.22	Average of absolute value of errors		1.11

The errors are calculated using the formula ${\text{Error}} = \frac{{{\text{FEM}} - {\text{DTM}}}}{{{\text{FEM}}}} \times 100$.

**Figure 3:**
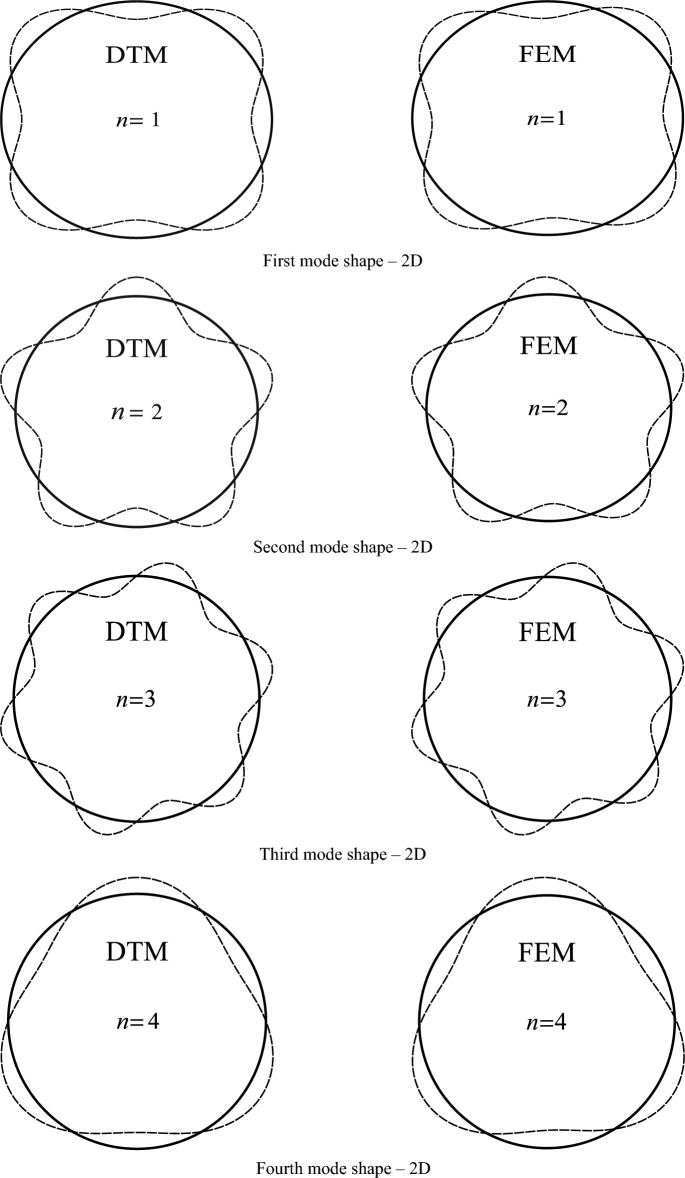
The first to forth 2D mode shapes of the closed cylindrical shell.

**Figure 4:**
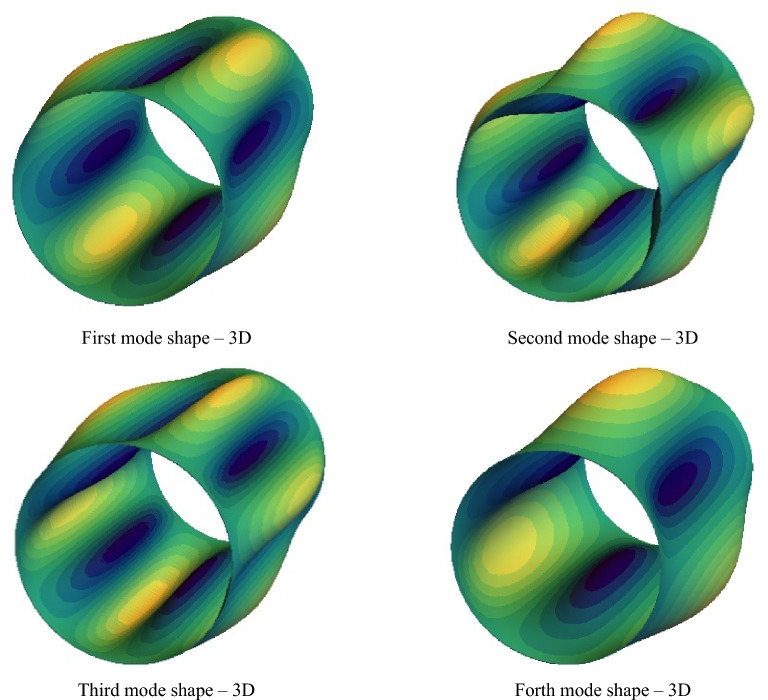
The first to forth 3D mode shapes of the closed cylindrical shell.

**Figure 5:**
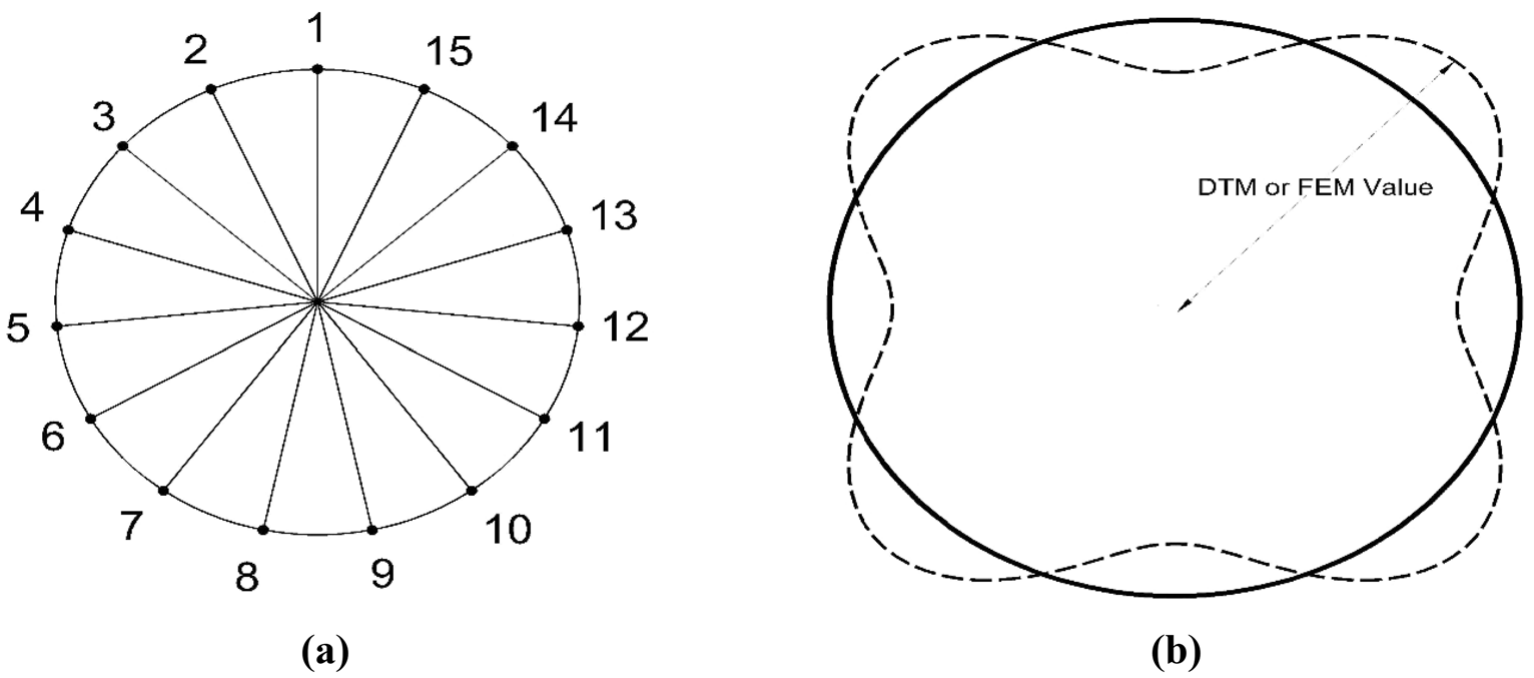
The definition of point and sections for 2D mode shapes comparison, (**a**) points numbering, (**b**) the value of the displacement.

By observing the results which are shown in Figs. 3 and 4, DTM is considered to be an exact method. The mode shapes are very similar in all of the outputs. The high accuracy of this method, which can be conducted from its meager percentage of error, can indicate the high ability of this method compared to the existing theories and even the capacity of DTM to get used instead. In the following, the parametric analysis is discussed (Fig. 5).

## Parametric analysis

**Figure 6:**
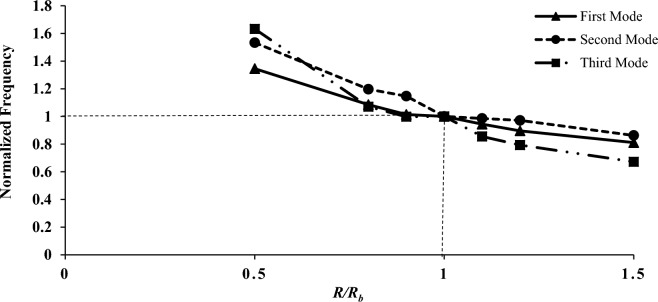
The normalized frequency as a function of shell radius.

**Figure 7:**
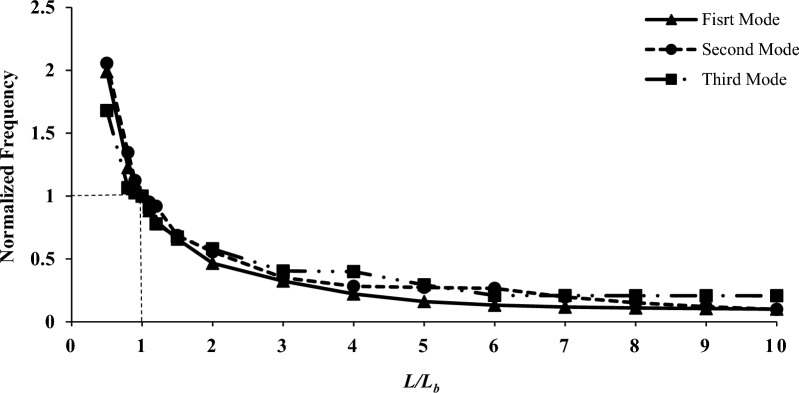
The normalized frequency as a function of shell length.

**Figure 8:**
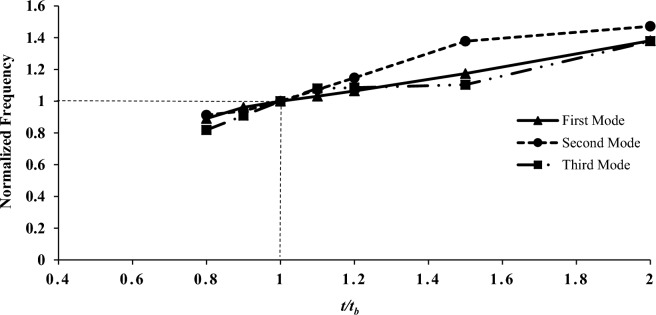
The normalized frequency as a function of shell thickness.

**Figure 9:**
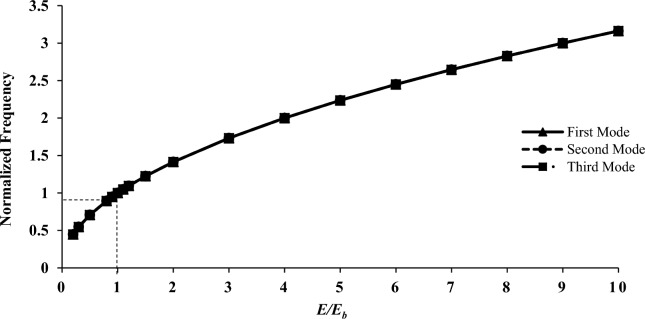
The normalized frequency as a function of shell elasticity modulus.

In this section, the effect of the mechanical and geometric properties of the shell on variation parameters is investigated. Among the parameters, modulus of elasticity, shell length, shell thickness, mode number change, and shell radius can be mentioned. For a more straightforward and more general application, the results data related to the process of mechanical and geometrical properties were normalized to the outcomes presented in the first to third modes, as listed in Table 5 (the base model, B). The support and other properties are similar to the previous modeling. Also, base parameters in all investigations are summarized in Table 2. Figures 6, 7, 8 and 9 present the assessment of mechanical and geometric properties in a dimensionless manner.

As shown in Figs. 6, 7, 8 and 9 the diagram of dimensionless frequency variation versus the dimensionless properties follows a specific trend. In all diagrams, the base frequencies are $F_{b1} = {224}.{43}\,{\text{Hz}}$, $F_{b2} = {2}32.18\,{\text{Hz}}$, and $F_{b3} = {2}99.30\,{\text{Hz}}$ for the first to third modes, respectively. It's worth noting that he frequency illustrated in the graphs is directly related to the modulus and thickness, while it has an inverse relationship with the radius or length.

Figure 6 is related to the investigation of the shell radius property, which is shown for modes numbers one to three. The base radius is $R_{b} = 0.175\,m$. It can be observed that the frequency decreases with the increase in radius. The frequency increases is significantly higher in lower radii. Additionally, in the third mode, more sensitivity to the radius change is observed which shows the increase in the influence of the radius with the increase of the mode number. It should also be noted that the trend in this parameter is almost linear.

Figure 7 is related to the frequency change in the geometric trend of the shell length with the base value of $L_{b} = 0.664\,m$. It is explicit that the frequency decreases as the length increases; however, the shape of the graph has taken a logarithmic curve. This means there is high-frequency sensitivity to change in low lengths and, conversely insensitivity in high lengths. It is in such a way that practically no change in frequency is observed for high lengths. Another noteworthy point in this figure is the closeness of the normalized frequency data in the same conditions and in the first to third modes. It should be noted that, similar to the radius parameter, the influence of the third mode is lower than that of the first and second modes, and this mode follows a different process after the transmission point (point 1).

Besides, Fig. 8 depicts the reverse trend of the length chart. In this diagram, the frequency increases with the increase of the shell thickness under the same conditions as other geometrical parameters. As observed, the trend is nearly linear. Unlike the previous two figures, the mode number did not influence the reversal of the graph after the convergence point 1, and the order of the values after this point remained unchanged.

Figure 9 displays the graph of changes in the modulus of elasticity versus frequency. Interesting points can be extracted from this chart. As can be seen, in all modes from the first to the third, the normalized frequency is exactly the same under the same conditions. Furthermore, there's a discernible increase in frequency with the rise in the modulus of elasticity. Another critical point is that the frequency increases as the modulus of elasticity increases; however, this trend follows a specific relation. The relation between these quantities is given by:

40 $$ \left( {\frac{E}{{E_{b} }}} \right)^{0.5} = \frac{F}{{F_{b} }} $$

## Conclusion

This study presented a method to analyze and investigate the natural frequency of cylindrical shells using the DTM for the first time, according to the author's knowledge. In this regard, Initiatives were made to prepare the equations to be solved by the DTM without changing or modifying the principles of the method. Moreover, owing to the novelty of using the method in cylindrical shells, and to demonstrate the accuracy of method, the hybrid finite element method was employed for validation. Key findings show inverse and exponential relationships between shell dimensions and frequency, with the dimensionless frequency directly related to the square root of the dimensionless modulus of elasticity.

Furthermore, the DTM is advantageous because it generates a significantly smaller system of equations compared to the Finite Element Method. This contributes to its computational simplicity and efficiency, making it a valuable tool for analyzing the free vibration of cylindrical shells.

## Data Availability

The data that support the findings of this study are available from Farzad Shahabian but restrictions apply to the availability of these data, which were used under license for the current study, and so are not publicly available. Data are however available from the authors upon reasonable request and with permission of Farzad Shahabian.

## List of symbols

$N_{xx}$: Normal stress resultant in axial direction
$N_{x\theta }$: Shear stress resultant in the $x - \theta$ plane direction
$N_{\theta \theta }$: Normal stress resultant in circumferential direction
$M_{xx}$: Stress couple resultant in axial direction
$M_{x\theta }$: Stress couple resultant in the $x - \theta$ plane direction
$M_{\theta \theta }$: Stress couple resultant in circumferential direction
$$x$$: Axial coordination
$R$: Cylindrical shell’s radius
$$\theta$$: Circumferential coordinate
$\varepsilon_{x}$: Normal strain in axial direction
$\varepsilon_{\theta }$: Normal strain in circumferential direction
$\varepsilon_{x\theta }$: Shear strain resultant in $x - \theta$ plane direction
$\kappa_{x}$: Strain couple resultant in axial direction
$\kappa_{\theta }$: Strain couple resultant in circumferential direction
$\kappa_{x\theta }$: Strain couple resultant in $x - \theta$ plane direction
$$u$$: Axial displacement
$$v$$: Circumferential displacement
$$w$$: Radial displacement
$\overline{U}$: Differential transformation of axial displacement
$\overline{V}$: Differential transformation of circumferential displacement
$\overline{W}$: Differential transformation of Radial displacement
$$\left\{ \sigma \right\}$$: Stress vector
$$\left[ P \right]$$: Elasticity matrix
$$\left\{ \varepsilon \right\}$$: Strain vector
$$E$$: Young’s modulus
$$t$$: Time
$$\upsilon$$: Poisson’s ratio
$h$: Cylindrical shell’s thickness
$L$: Cylindrical shell’s length
$$\mu$$: Poisson’s ratio
$$\omega$$: Shell’s frequency
$\rho$: Cylindrical shell’s density

## Code availability

Mathematica version 12-1 coding software is used in this article and the URL link to access the code is given below. https://drive.google.com/file/d/1Bl3nsNXCcB4e2Kp0jxHu8ovuo5j12vsy/view?usp=sharing.
